# Analysis of Interurban Mobility in University Students: Motivation and Ecological Impact

**DOI:** 10.3390/ijerph17249348

**Published:** 2020-12-14

**Authors:** Javier Cruz-Rodríguez, Amalia Luque-Sendra, Ana de las Heras, Francisco Zamora-Polo

**Affiliations:** Departamento de Ingeniería del Diseño, Escuela Politécnica Superior, Universidad de Sevilla, C/Virgen de África, 7, 41011 Sevilla, Spain; javicrurod@gmail.com (J.C.-R.); amalialuque@us.es (A.L.-S.); adelasheras@us.es (A.d.l.H.)

**Keywords:** mobility, smart campus, means of transport, students, university, SDG 7, SDG 11, SDG 13

## Abstract

The management of mobility in large cities is a complex issue of great interest due to its economic, social, and environmental impact. In this work, the interurban mobility of engineering students from two campuses of the University of Seville is studied. Specifically, this work carries out an analysis of the preferences of students in terms of mobility to their study centres and determines the environmental impact of such mobility in terms of kg of CO_2_ per student. Three constructs can be found to describe the motivation for their choice of transport: those related to comfort and speed, those related to sustainability and price, and those related to safety. Based on the responses obtained, groups of students are established that enable the design of specific actions in accordance with each of the profiles. From the analysis of the results obtained, recommendations are made for policymakers, and a reflection is given on the impact of the COVID-19 pandemic on this issue.

## 1. Introduction

Mobility management in cities constitutes a major challenge [1,2]. There has been a rapid increase in city populations. In 2007, 50% of the world’s population lived in cities, and according to the United Nations’ Habitat program, it is estimated that by 2050, two-thirds of the world’s population will live in cities [3].

The United Nations has proposed an agenda that seeks to promote sustainable human development, the well-known Sustainable Development Goals (SDGs) [4]. The SDGs were approved in the year 2015 by the General Assembly of the United Nations [4] and represent a first-level challenge for all countries. To achieve these goals, the participation of all actors is required, including governments, civil society, enterprises, and non-governmental organisations [4,5,6]. The 2030 Agenda must be understood in a joint way, since the SDGs are interconnected with each other [7,8,9].

Institutions of higher education play a fundamental role in achieving these SDGs [7,8,10,11,12,13]. In the exercise of teaching, research, and the transfer of results to society and governance, the university can provide a catalyst for social change [10,14,15]. Despite this importance, sustainability on university campuses still has plenty of room for improvement [16].

In previous work, we reflected on how the university can contribute towards the fulfilment of the Sustainable Development Goals through teaching [8,9] or research [17]. In this case, we want to reflect on how the university can contribute to sustainable development with campus operations. We are interested in ascertaining the means of transport used by students in their transportation to the university, the reasons for their choice, and the prospects for change in the future. Engineering is an area of special interest for the achievement of sustainable development in general [1,18,19,20] and the Sustainable Development Goals in particular [20,21,22], and for this reason, engineering students are those analysed in this study.

Previous work has reflected on the importance of using sustainable means of transport by the university community. In this respect, Mateo-Babiano et al. (2020) analysed the use that Filipino university students made of a system of shared bicycles [18], and Cattaneo et al. (2018) analysed the attitudes of university students towards various sustainable modes of transport [23]. In their work, the researchers revealed that the particularities of university campuses condition the type of student mobility. The use of transport in a Qatar University was carried out as a previous step for a sustainable mobility plan [1]. That work showed that a high number of members of the university community came to the university in their own cars and showed the importance of infrastructure in promoting mobility of a more sustainable nature. The motivation for walking on campus was analysed in students from Malaysia [24]. A study at a Greek university showed that a large majority of trips were made using public transport well in excess of those on foot or travelling in their own vehicles [25]. The motivation for the use of sustainable means of transport in the European Union was studied by Heras Rosas and Herrera (2019) [26].

**Figure 1:**
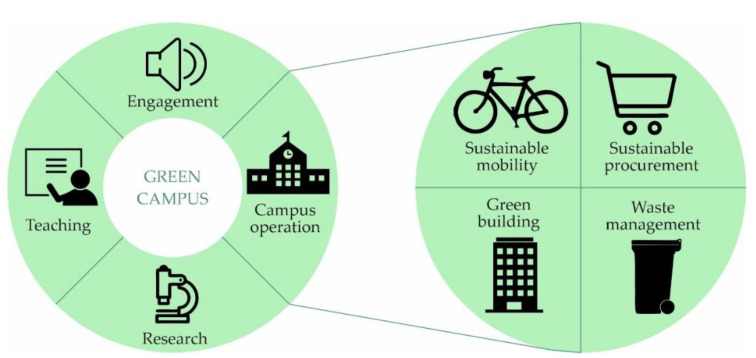
Green campus framework. Source: Authors’ own. Based on Fissi et al., (2021) [27].

Fissi et al. (2021) consider mobility as part of a strategy for the construction of green campuses [27]. In this vein, they consider that the university can contribute towards sustainability from four fundamental facets: teaching, research, promotion of sustainability among members of the university community (engagement), and campus operations. It is precisely in this last dimension where they consider mobility through the promotion of cheaper and sustainable means of transport [13,27] (Figure 1). The promotion of more sustainable modes of transport has effects on the environment, on the economy, and on the health of users [28].

In previous work, the analysis of the life cycle of universities has been carried out [19,29]. Universities are very complex entities, and there are numerous factors that must be considered when evaluating the environmental impact of their activity [19]. In this respect, transport and mobility are considered in the majority of the studies carried out regarding the analysis of the life cycle of the university [19].

Several articles include recommendations for policymakers. Azzaly and Abdel Sabour [1] propose a framework for the improvement of transport at Qatar University. Based on an analysis of transportation needs, they propose a mobility plan for the university that includes actions in infrastructure, transportation, and culture [1]. The evaluation of the current situation and the knowledge of the opinions of the users of the means of transport are fundamental for the definition of public policies that promote sustainable mobility [23].

In Spain, the sustainability commission, created within the Conference of Rectors of Spanish Universities, is responsible for evaluating environmental policies of Spanish universities and for promoting actions to reduce the environmental impact created by universities [30]. It has several working groups: Evaluation of University Sustainability, Environmental Improvements in University Buildings, Sustainable Mobility, and University Urbanism [30]. Ascertaining the opinions of students becomes crucial when promoting policies to promote sustainability on university campuses [15].

The University of Seville (Spain) is an institution founded in 1505 and hosts 70,900 undergraduate, graduate, and doctoral students in its 32 owned and affiliated centres [31].

The University of Seville has 13 campuses distributed throughout different areas of the city. It is made up of 56 buildings, including faculties, libraries, administration services, sports facilities, and general services.

**Figure 2:**
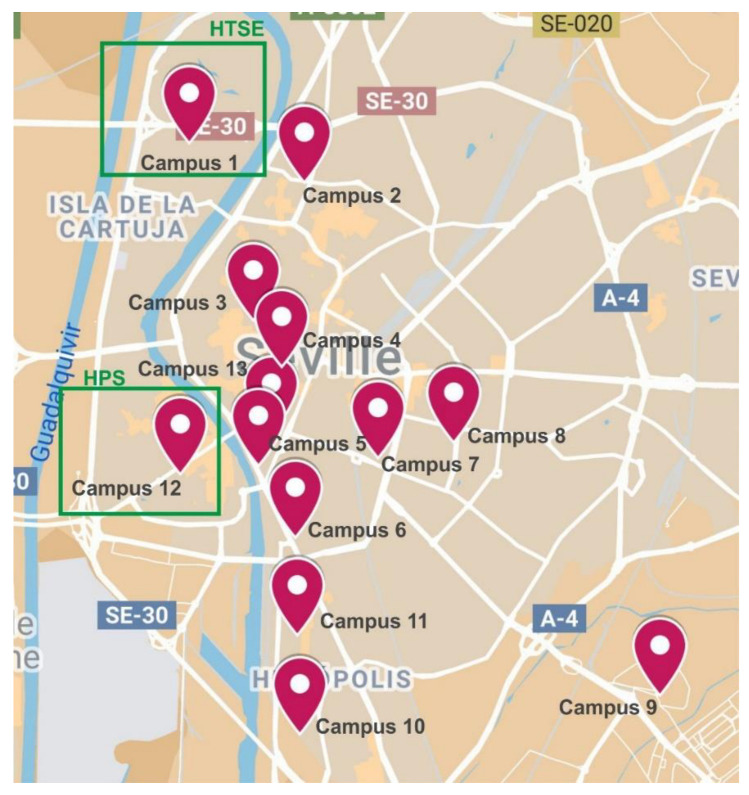
Campus distribution of the University of Seville [32,33].

The areas in which the 13 campuses are located are all accessible by urban transport (Figure 2). Several of these campuses are located in the city centre and therefore enjoy better connections with public transport (train, metro, bus, bicycles, etc.). However, they also suffer from many more restrictions on car use due to the reduction in parking opportunities and to policies aimed at reducing the number of cars and at promoting pedestrianisation in downtown areas of the city. In contrast, those campuses located in the areas outside the city centre have a good public transport structure as well as an increase in the possibility of access to public parking. This situation also favours the use of private cars due to the decrease in services and transport combinations available.

The case that concerns us involves campuses 1 and 12 that together represent the university areas covering the field of engineering. The centres that will be analysed in this paper are the Higher Technical School of Engineering (HTSE) (Campus 1) and the Higher Polytechnic School (HPS) (Campus 12).

All areas of the city, and therefore all its campuses, are easily accessible through public transport throughout the week, since the city grants extended hours for the use of all of these means of transport. However, regarding the use of cars, from Monday to Saturday, there are paid outdoor parking areas throughout the city that make it difficult to park cars that do not belong to the residential area, especially in downtown areas and neighbouring annexes. The Higher Polytechnic School (HPS) campus is located in a neighbourhood attached to the downtown area of the city, and although it has high accessibility for all means of public transport, vehicle parking remains very limited owing to the overcrowding of residential vehicles. For the Higher Technical School of Engineering (HTSE) campus, the opposite is true; it is not located in a central or adjacent area and contains no residential buildings, and hence parking is free and more abundant. This situation causes the most commonly used means of transport for this campus to be the private vehicle.

The Higher Polytechnic School will soon be moved to the Scientific and Technological Park “Isla de la Cartuja” (Campus 1) in new facilities.

The objectives of this work are:

1. To analyse the preferences of students in terms of mobility to their study centres.
2. To determine the environmental impact of such mobility in terms of kg of CO_2_ per student.

The structure of the article is as follows: in the next section, the methodology is described. For this purpose, the questionnaire employed, the sample analysed, and the statistical methods applied are all detailed. In Section 3, the results are presented: firstly, the mobility patterns of the two schools analysed are described and the reasons expressed by the students for their choice of the means of transport and the evaluation of these are then analysed. The alternative means of transport are subsequently analysed, as are the CO_2_ emissions emitted by each of the groups. In Section 4, the results are discussed in comparison with other findings in the literature. Finally, the conclusions of the work are presented together with recommendations for policymakers, and the possible influence of COVID-19 is described.

## 2. Methodology

### 2.1. Questionnaire

A questionnaire was developed for this study. In order to create this questionnaire, a bibliographic search was made of similar studies [1,18,23,24,25]. A brainstorming methodology was employed, in which 27 people participated, to determine the constructs that students would use to justify their choice of means of transport. Following the affinity analysis, it was decided that the descriptive terms that should be considered are (a) Dynamic, (b) Availability, (c) Freedom, (d) Comfort, (e) Environmental Quality, (e) Price, (f) Innovative, (g) Non-Polluting and (h) Safety. A total of 3 experts evaluated the questionnaire. They provided certain minor modifications in the implementation of the questionnaire.

The questionnaire is structured in 3 sections (Appendix A). The first is related to the sociological data of the students (gender, age, degree) and their habitual residence. The second section asks about their most common means of transport, as well as the reasons for choosing this means of transport, and an assessment of the current means of transport and its future viability. In this section, the students were asked about the number of daily trips and the number of days per week they attend the university. Finally, the last section asks whether students are willing to change their means of transport, to which means of transport they would change, and the emotions/feelings that this new means of transport would provoke in them.

### 2.2. Sample

Due to the exploratory character of our study, a non-deterministic sample was carried out. The students interviewed come from either of the two centres of the University of Seville that offer engineering studies.

Specifically, 289 students from the Higher Technical School of Engineering (HTSE) and 288 students from the Higher Polytechnic School (HPS) were randomly selected from among the total number of students in each centre.

The average age of the students is 21.25 years for undergraduate students and 23.8 years for Master’s students. Ninety percent of the students are currently taking undergraduate degrees, and 10% are taking graduate degrees. Of the students surveyed, 72% are male and 28% are female. These percentages differ slightly from the ratio of men to women in engineering studies across Spain [34] and similar to other previous studies [35]. It should be borne in mind that this difference is predominantly due to the degrees of industrial design engineering and chemical engineering, whereby the percentage of female professionals in Seville remains higher than the average in engineering studies across Spain.

The questionnaire described above was provided in electronic format using the Google Form tool, similar to other previously published studies [7,36,37]. The advantages of the electronic distribution of a questionnaire have been described in previous work, and these include the fact that anonymity is guaranteed and that the transcription of the results is easier than paper-based questionnaires [7,35]. The questionnaire was supplied between January and February 2020, previous to the first lockdown triggered by the COVID-19 disease. In terms of ethical considerations, participants were informed about the purpose of the study, responses were anonymous, and participation was voluntary.

### 2.3. Statistical Analysis of the Results

For the study of statistical results, the Statistical Package for Social Science (SPSS) (IBM) software version 26 for Windows (IBM, Chicago, IL, USA) was employed [38].

A descriptive analysis of the results obtained was first carried out in order to verify the difference between two populations. The existence of normality (Kolmogorov–Smirnov test and Shapiro–Wilk test) and homoscedasticity (Levene test) was also verified in order to use ANOVA. In the cases where it has been possible, the hypothesis contrast for ANOVA averages has been applied while considering a significance of 5%. Where this was not possible, non-parametric contrasts (Mann–Whitney U test) with a significance of 5% were used.

A factorial analysis was carried out in an effort to group the students’ motivation when choosing the means of transport. The method used was that of principal component analysis. The element extraction method is based on the eigenvalues greater than 1. The maximum number of iterations for convergence was 100. The Varimax method was selected to obtain the rotated component matrix. To determine the relevance of the application of this analysis, the Bartlett sphericity test was performed, and the Kaiser–Meyer–Olkin (KMO) sample-adequacy measurement was calculated. 

The evaluation of the constructs obtained through factorial analysis was analysed by determining Cronbach’s alpha coefficient [39].

For the establishment of the user profiles, an ANOVA analysis has been used in order to deduce which survey questions to use in the User Test. The Levene test, Kolmogorov–Smirnov test, and Shapiro–Wilk test were analysed to ascertain which questions were available for their integration into or removal from the User Test in accordance with the parametric tool ANOVA Analysis. For two questions, the Kruskal–Wallis test was applied, since these failed to correspond to a parametric tool, and hence, a non-parametric tool was needed.

Once the previous analysis had been carried out, it was time to perform a hierarchical cluster analysis in order to ascertain the number of questions in the cluster. First, a dendrogram was developed, and subsequently, a sufficiently representative number of clusters were chosen based on the number of iterations of SPSS required in order to attain an optimal number of profiles. In this way, the distance of the dendrogram is obtained.

Finally, to improve the resolution of the results, the non-hierarchical analysis, K-means cluster, was used. Since the ideal number of clusters was known, it was possible to determine the table of Final Cluster Centres, how many respondents belonged to each cluster, and the scatter plot.

### 2.4. Determination of the Ecological Footprint

In order to determine the ecological footprint, the distance in kilometres between the student’s habitual residence and the educational centre was calculated. For this purpose, the computer tool Google Maps [32] was used. In order to determine the amount of CO_2_ emitted per week, both the number of trips made per week and the degree of occupation of the vehicle were considered. The emission factors of each of the types of vehicles were obtained from previously published work (internal combustion engine (ICE) car [40], hybrid car [41], internal combustion engine (ICE) motorcycle [40], electric motorcycle [42,43], electric scooter [43,44], bus [40], train [41], tram [41], and metro [41]).

The work has considered the level of occupation of the cars. Table 1 shows the amounts of CO_2_ (in kg) emitted per km for ICE and hybrid cars according to the percentage of occupation.

## 3. Results

The results of the questionnaire are set out below. Firstly, the distribution of the most common means of transport is presented, and subsequently, the reasons for this choice are analysed, as well as the assessment of the means of transport. From the results obtained in the users’ answers, different user profiles are then obtained. Finally, the impact of the CO_2_ footprint of the analysed sample is calculated.

### 3.1. Means of Transport Used

**Figure 3:**
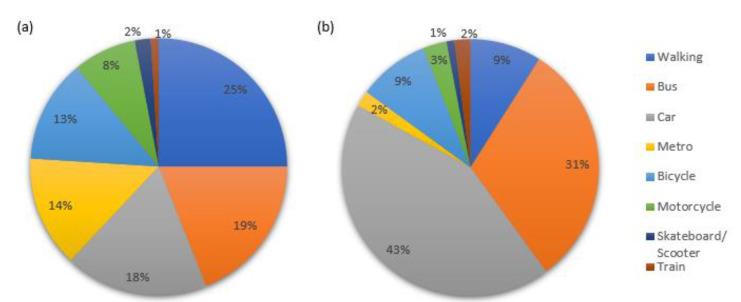
Means of transportation most frequently used by students of (**a**) Higher Polytechnic School (HPS) and (**b**) Higher Technical School of Engineering (HTSE).

From the analysis of the results of Question 6 (Q6), various patterns in the mobility of students from the analysed centres can be found. Figure 3 shows the means of transportation frequently used by students at each of the university centres.

As can be observed from Figure 3, there is a clear difference between students’ mobility preferences. The HPS students often come to their study centre on foot (25%), by bus (19%), or to a lesser extent by car (18%) and metro (14%). The most frequent means of transportation for HTSE students is by car (43%) followed by bus (31%), bicycle (9%), and on foot (9%). Previously published studies have found differences between mobility patterns in students from different campuses of the same university [23].

### 3.2. Reasons to Use the Means of Transport and Valuation

Table 2 shows the average student responses for each of the questions Q7–Q14. These questions focus on the students’ motivation for their choice of transportation. According to the total number of students, the means of transport that are considered to be the fastest (Q7) are the scooter, the motorcycle, and the bicycle. At the other end of the scale are the bus and walking. In terms of the availability of transportation (Q8), the best-rated means of transportation are bicycles and scooters. In terms of freedom (Q9), the most sustainable means of transport, such as walking, bicycles, and scooters, achieve the highest scores, with collective means of transport such as the metro, bus, and train achieving the worst scores. Regarding comfort (Q10), students give the highest score to the car and the scooter. Items 11, 12, and 13 are clearly correlated. Students value walking, cycling, and skateboarding as sustainable and cheap means of transport. In terms of safety, students consider walking the safest form of mobility, with motorcycles, bicycles, and skateboards among the most unsafe. As indicated in the Methodology section, the survey was provided prior to the World Health Organisation’s declaration of the COVID-19 pandemic (11 March 2020) [45], and hence, the values presented here do not reflect the impact that the COVID-19 has exerted on public transport.

In order to ascertain whether there are any differences between students from the separate study centres (HPS and HTSE), a normality analysis was first performed to verify that the ANOVA test can be used. Table 3 shows the results of this normality test for all the samples and indicates whether the test is the Kolmogorov–Smirnov or Shapiro–Wilk test. Those samples that have a p-value higher than 0.05 are marked with an *. For normal samples, the ANOVA test was employed to compare averages of independent samples while for non-normal samples, the Mann–Whitney U test was used. In the table, those cases in which significant differences have been found are marked with an *.

As can be observed, no significant differences could be demonstrated in the tests performed.

A factorial analysis was performed with the help of the SPSS software. The results of the KMO test and the Bartlett sphericity test are shown in Table 4. The value of the KMO test is not very high: most authors recommend a value above 0.7 [46,47]. However, the results of this analysis can provide an idea of the relationship between the items in the survey.

Table 5 shows the eigenvalues associated to each of the main components together with the percentage of variance explained by the components and the percentage of accumulated variance.

The previous results show that with the first three components, 73.631% of the variance is explained by the model. Table 6 shows the values of the rotated components with the main component method used as the extraction method, and the Varimax rotation method with Kaiser normalisation. The solution was obtained after four iterations.

The factor analysis indicated that the three factors obtained were mainly related to the following items: Q11–Q13 (C1), Q7–Q10 (C2), and Q14 (C3). The results obtained are very interesting. The first factor is associated with sustainability and economy, the second factor is associated with comfort in use, and the third factor is associated with safety.

In order to corroborate that these items can constitute independent constructs, Cronbach’s alpha coefficients were calculated for each of these constructs, and the following values were obtained: 0.765 (C1) and 0.862 (C2): both values higher than 0.7. Hence, these can be considered as independent constructs [7,9,39,48]. These results are of interest because the factors are grouped and therefore allow us to look for relationships with other variables and to establish strategies to influence these variables.

**Figure 4:**
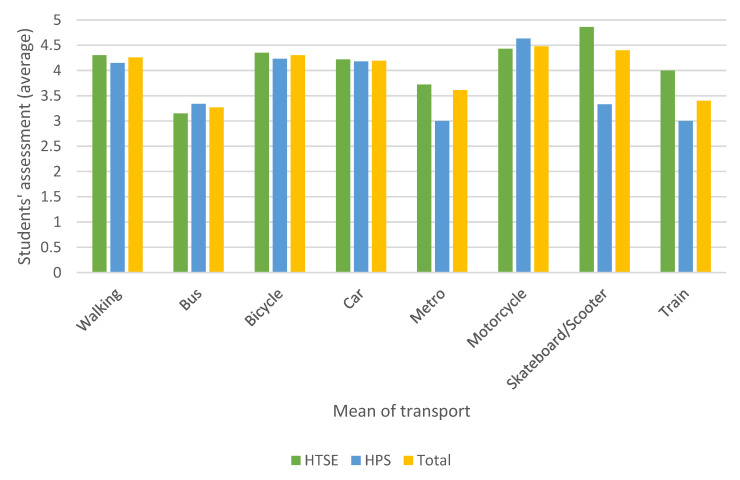
Average of students’ assessment of the most frequently used modes of transportation (Q15).

**Figure 5:**
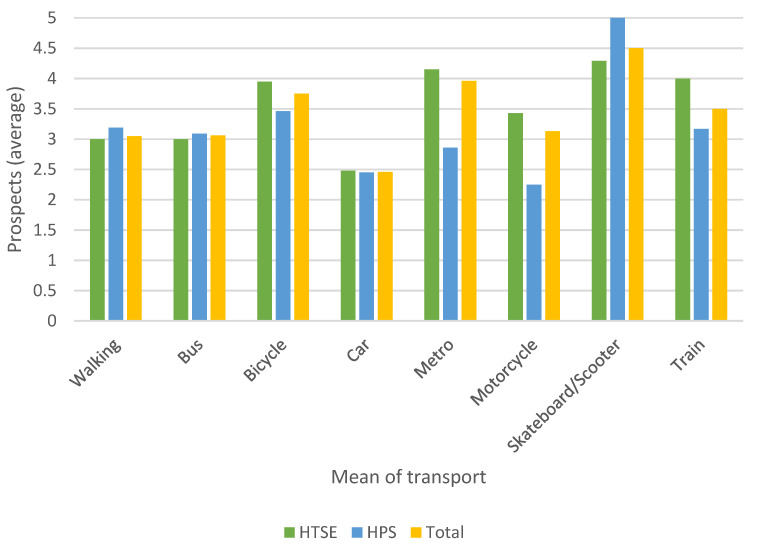
Average of prospects of the selected mode of transport (Q16).

Figure 4 and Figure 5 show the evaluation of each means of transport chosen by the students (Q15) and the viability of this means of transport for the future (Q16).

In the evaluation, motorcycles, scooters/skateboards, and walking attain the best ratings. On the other hand, collective passenger transport (metro, bus, and train) is among the least-valued means of transport. As regards prospects, the students do not show a clear commitment to maintaining the means of transport used today in the future. The scooter, metro, and bicycle are the most highly rated in this respect. It is striking that the car, a means of transport frequently used today, reaches a very low value in the score of this item. This result shows the possible change in the energy model that we are moving towards, which includes questioning the use of ICE vehicles and committing to vehicles of a more sustainable nature.

The differences between university centres for questions Q15 and Q16 were analysed similarly to the motivation expressed in Table 3, and significant differences were found only for item Q16 in the metro and for the motorcycle by non-parametric tests.

The above results seem to show that collective public transport (CPT) is valued differently from other means of transport (NC). To verify whether an ANOVA test could be used, a normality test was first performed on the Q15 and Q16 item scores in accordance with the Kolmogorov–Smirnov test. Table 7 shows the results of the normality test. As can be observed, it cannot be stated that the data follows a normal distribution, and therefore, the ANOVA test cannot be applied. As an alternative, the Mann–Whitney U test was used, and the results are expressed in Table 8. From these results, it can be concluded that there are significant differences between the assessments that students make for questions Q15 and Q16 depending on whether the means of transport is collective or not. The average rating of the non-collective transport amounted to 4.26, in contrast to that of the collective public transport, which amounted to 3.23. Regarding Q16, the collective means of transport obtained an average score of 3.29, while the remaining means of transport gained a score of 2.94.

These results are contradictory to those stated in reference to the use of the means of transport. Public transportation is utilised by many students; however, it receives a low evaluation. On the other hand, students consider that these means of transport are in fact the future. The reasons that justify these differences should be investigated in future research. In previous work, this use of public transport has been argued as being due to the low percentage of students who have their own car [25]. Furthermore, these results should be tested in the current pandemic situation.

### 3.3. Alternative Means of Transport

In order to analyse this aspect in greater depth, questions 17–19 address a possible change in the most frequent means of transportation by students. A very high percentage of students (44.34%) said they would change their means of transportation, 27.30% said they might change, and 28.35% said they would not. Table 9 shows the results based on the means of transport currently used. These results express the opinion of the students of both centres.

According to the above results, a substantial percentage of bicycle users and people who go to university on foot show that they are loyal to these means of transport and do not want to change. On the other hand, it should be noted that a very significant percentage of users of the most widely used means of transport (the car) say they want to change to another means of transport.

**Figure 6:**
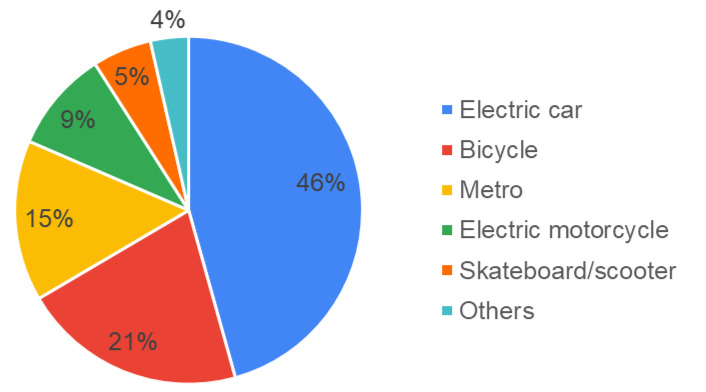
Alternative means of transport.

Regarding the means of transportation they would choose as an alternative, Figure 6 shows the means of transportation chosen by the students as a percentage. As can be observed, most students would choose the electric car, followed by the bicycle, the metro, and the electric motorcycle.

**Figure 7:**
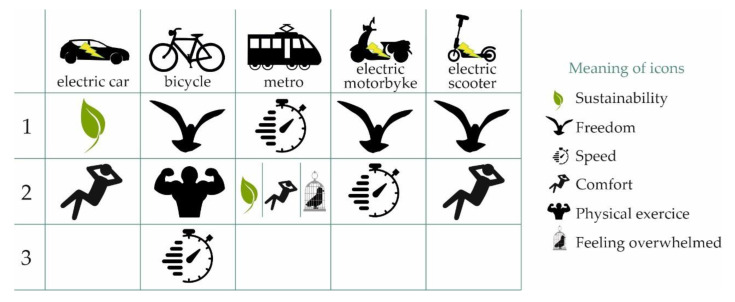
Emotions and feelings associated with each of the alternative means of transport.

The analysis of the open responses in which students express their feelings/emotions provoked by alternative means of transport has proved highly informative (Q19.1). In this respect, students associate the electric car with sustainability and comfort; the use of the bicycle with freedom, the possibility of physical exercise, and with speed; the use of the metro with various concepts such as speed and aspects such as sustainability and comfort, while others relate it with a certain lack of freedom. The electric motorcycle is associated with the concepts of freedom and speed, and the electric scooter is associated with the concepts of freedom and comfort. Figure 7 reflects the sensations that each of the alternative means of transport produce in the users.

Regarding the reasons that would lead them to change to an alternative means of transportation, among the students that are not fully decided are those expressed in Table 10.

As can be seen in the above results, the students value the speed, sustainability, price of the new means of transport, and comfort, in that order. These results can be utilised to promote more sustainable means of transport through institutional advertising campaigns.

### 3.4. User Profiles

A K-means cluster, which is a non-hierarchical analysis based on the clusters’ number obtained from the dendrogram [49] of the hierarchical cluster analysis, was performed to obtain the different user profiles. The distance taken in the dendrogram was 7.5 since, in twelve SPSS iterations in hierarchical cluster analysis, this converges into seven clusters, and hence 7.5 was the distance where the seven clusters were consolidated.

K-means is used because it is a non-hierarchical reassignment method based on the mean; therefore, if a hierarchical analysis is performed first to estimate the clusters’ number first, then K-means can better organise items.

Hierarchical grouping is capable of setting the number of clusters on its own; for this reason, they can be used in an exploratory way and subsequently apply a non-hierarchical analysis with the cluster number already set.

To this end, the data used includes that from questions Q6, Q15, Q16, and Q17, and the average of each respondent’s constructs. Using Table 11, seven profiles were selected that incorporate most of the users in the sample.

The translation of this table is commented in the following points:

- Transport: 1 = walking, 2 = bus, 3 = bicycle, 4 = car, 5 = metro, and 8 = motorbike
- Satisfaction: (0.0–2.5) = low, (2.5–3.5) = neutral, (3.5–4.5) = high and (4.5–5.0) = very high.
- Future: (0.0–2.0) = very low probability, (2.0–2.5) = low, (2.5–3.5) = neutral, (3.5–4.5) = high, and (4.5–5.0) = very high probability.
- Sustainable change: 1 = No and 2 = Yes.

**Figure 8:**
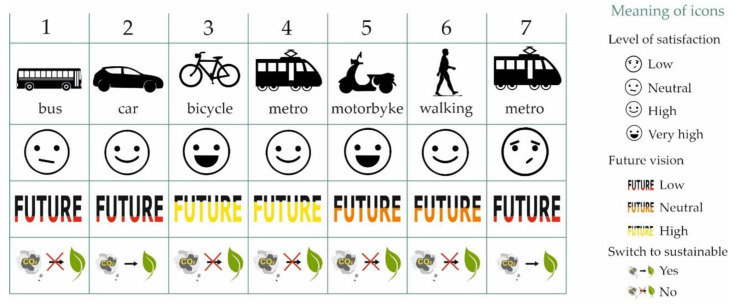
Summary of the characteristics of the clusters obtained.

This information can be expressed in a more visual way, as shown in Figure 8. This figure represents the current means of transport used by the students; the degree of user satisfaction; the degree to which the students consider that the means of transport will be used in the future, which is given in three levels (green: high, yellow: neutral, red: low); and whether the participants in the user profiles consider it necessary to change to a more sustainable model.

The main characteristics of the clusters obtained are described below.

The first group (83 students) is made up of users who choose the bus as their means of transportation. Their rating is not very high; they probably use this means of transport because they have no other option. They consider that, in the future, this means of transport will evolve towards a less polluting collective means of transport such as the metro or the electric bus. They consider that the mode of transport is fairly sustainable because it is a collective mode of transport.

The second group (145 students) comprises students who regularly use cars as a means of transportation. The students show a high degree of satisfaction. The students are aware that the ICE car is source of pollution. Most of the users would change their cars for an electric vehicle.

The third group is formed of users who travel by bicycle (90 students). They show a very high degree of satisfaction with their use, consider it to be a sustainable means of transport, and would not change their use for another means of transport.

Fourthly, the cluster is made up of students satisfied with the use of the metro (50 students), who consider that the number of metro lines should be increased and thus become a more viable means of transport in the future. They are satisfied on average with this means of transport.

Motorcycle users (39) are very satisfied with their means of transport. They feel that perhaps in the future, this means of transport will evolve towards the electric motorcycle. They would not change their current means of transport.

The sixth group consists of students who prefer to walk to class (158). They are satisfied with their means of transport. However, they doubt whether it will be a viable mode of transport in the future. In principle, they do not want to change their mode of transport.

Finally, the seventh group is made up of metro users (11) who are dissatisfied with the service. They feel that public transport has many drawbacks and will not be a viable option in the future. They would choose the car as an alternative means of transport.

Table 12 shows the number of users associated with each cluster, and Figure 8 provides a summary of the results obtained.

**Figure 9:**
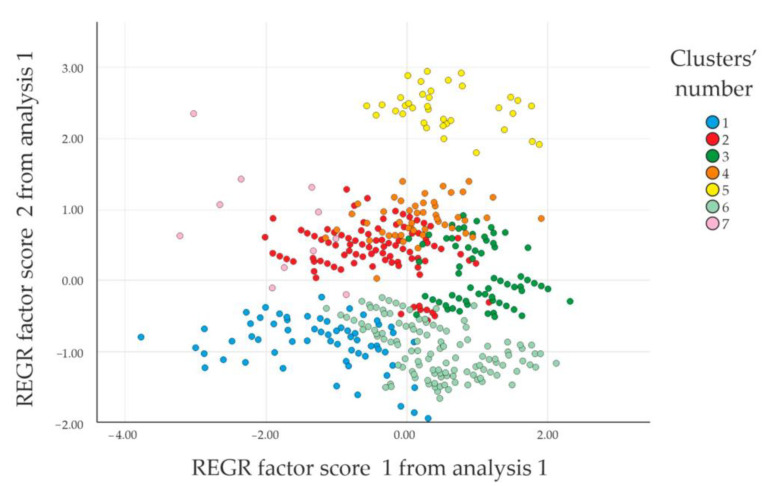
Scatter plot (REGR: regression).

In conclusion, Figure 9 shows the scatter plot of the clusters.

### 3.5. CO_2_ Emissions

**Figure 10:**
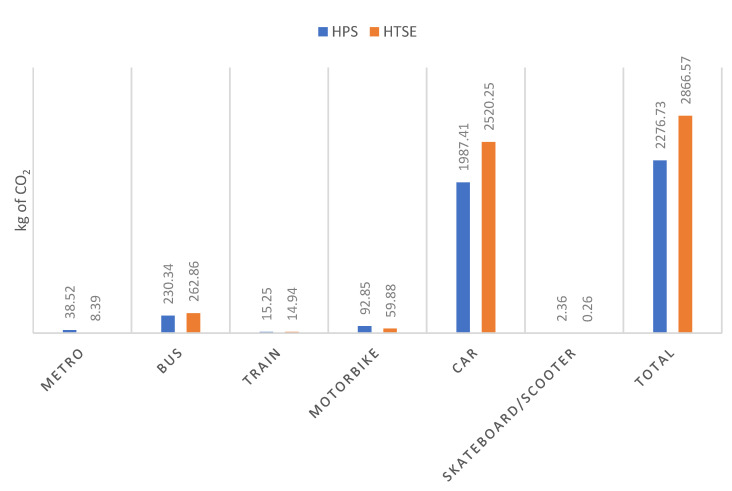
CO_2_ emissions/week of the samples.

Figure 10 shows the weekly CO_2_ emissions by each means of transport for student samples from each of the educational centres.

From Figure 10, it can be inferred that most of the total emissions come from the use of the car as a means of transport. If the CO_2_ emissions are expressed in terms of the number of students interviewed who have provided their address in an appropriate way, the CO_2_ emission of the student transportation to the HPS is 8.36 kg CO_2_/week/student, while HTSE produces 10.54 kg CO_2_/week/student. The values of one centre are 26% higher than those of the other centre. This may be justified based on the location of the buildings.

## 4. Discussion

The results shown in this work are in line with the Mobility Plan of the city of Seville [50]. According to this document, 8.8% of journeys in Seville are due to study trips, of which most are made on public transport (43.4%), 24.1% of the trips are in a private vehicle, 21.6% are made on foot, 7.7% are made by bicycle, and 3.2% are made by other means. Regarding the districts in which this work is framed, the Seville Mobility Plan [50] establishes that most journeys to and from “La Cartuja” (HTSE) are made by car or motorcycle (54.2%), by bus (18.8%), and by bicycle (11.9%). In the case of journeys made to or from “Los Remedios” (HPS), 35.5% of the trips are made by ICE vehicle, 31.5% are made on foot, and 22% are made by bus. The results obtained in our study probably vary due to the lower purchasing power of students compared to that of other user profiles, such as professionals.

**Figure 11:**
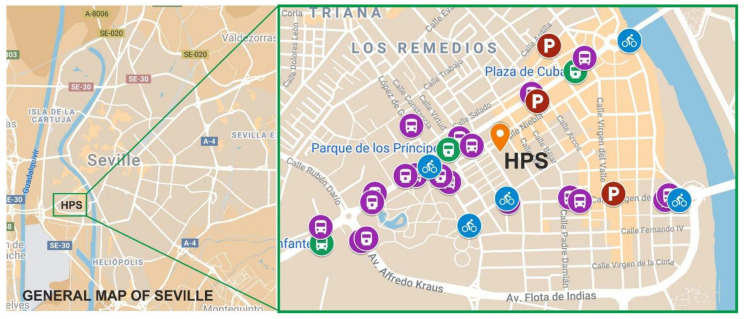
Georeferenced analysis of the HPS campus [32,33].

**Figure 12:**
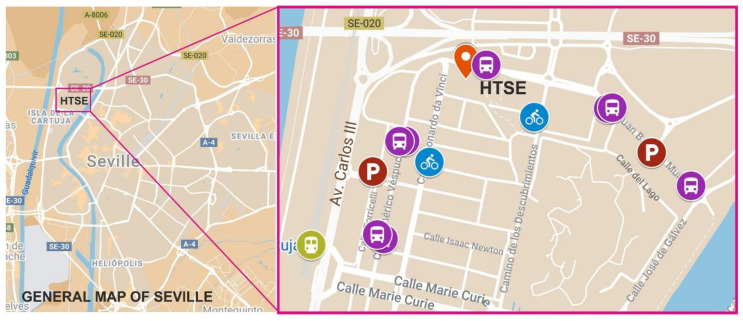
Georeferenced analysis of the HTSE campus [32,33].

In the following figures (Figure 11 and Figure 12), an analysis of both campuses and their associated transportation structures is laid out in order to clarify the situation of both campuses.

Figure 11 shows the campus to which HPS belongs. It is in the “Los Remedios” neighbourhood, attached to the city centre, which is a highly residential area. The buildings have an average of four to five floors and, according to the 2019 city census, it houses a population of 25,038 inhabitants. For this reason, this area enjoys many services and an extensive public transport infrastructure, but it also has a limitation in the number of parking spaces for vehicles, since those that exist are largely paid. Within the public transport network, there are 3 metro stations, approximately 15 bus stops with approximately 12 bus lines that connect with various parts of the city, 5 municipal bicycle rental stations within a radius of 1 km of the HPS, numerous rental motorcycle parking areas, 1 paid parking esplanade at a reduced price attached to the metro station that connects to the outskirts of Seville, and 3 paid car parks.

Figure 12 shows the location of the campus where the HTSE is located. As indicated, this campus is in a neighbourhood far from the central areas of the city, where the universal exhibition was held in Seville in 1992. Since 1993, it has become a business area, far from the residential areas of the city and lacking services. It is connected through public transport (bus, suburban train whose station is 1 km away, and bicycle rental network), although it has no metro station (the closest station is 4.5 km away). There are a dozen bus stops where the two main circular lines of the city connect, two bicycle rental stations, two paid parking lots, extensive free parking throughout the urbanised areas of the neighbourhood, and a train stop connecting to the outskirts that connects with other areas of the city and neighbouring towns. As it is not a residential area, it has a greater area of parking for vehicles, although this unleashes a situation of isolation, since it is too far to arrive on foot and requires the use of transport, thereby increasing the use of private vehicles.

The city’s urban transport network is committed to reducing pollution and the use of renewable energy. The metro network is supplied by 100% renewable sources. Likewise, since the introduction in the city of its single operational metro line, it has progressed from 20.3 to 13.9 gigawatts/hour of consumption. By analysing the data of the last seven years, the average consumption per user has decreased from 1.44 to 0.80 kilowatt hours (kwh).

As for the bus network, it has a fleet of Compressed Natural Gas buses. The use of natural gas in urban transport provides considerable environmental benefits compared to the use of traditional fossil fuels, which highlights the zero emission of solid particles and SO_2_, as well as the significant reduction of NO_x_ and CO emissions; a reduction is also achieved in CO_2_ emissions. With this reduction in emissions, the quality of the city’s environment has improved. Since the project began in 2006, the number of buses with this propulsion system has increased from 36 units (10% of the fleet) in 2006 to 275 units today (67% of the fleet).

Likewise, in the last 4 years, a network of electric rental motorcycles has been established in the city that, with more than a thousand motorcycles divided into several companies that currently have headquarters in Seville, offers citizens a simple, fast, and sustainable way of moving around the city.

From the literature review carried out, the link between the results obtained herein and previous research is sought. In this work, the differences due to location have been appreciated and, in addition, the hypothesis that the type of mobility varies according to the location of the university centre is verified. This is consistent with previous studies published in the literature [23].

In a study conducted at Qatar University [1], 69% of female students came to campus by car, 9% came by taxi, 1% came by carpool, and 21% came by other means of transportation (largely by bus). In the case of male students, 86% of the students had their own car, 8% caught a taxi, 6% travelled in a car with friends, and these male students did not report using public transport. A study carried out at the University of Bergamo [23] found that 77% of students used sustainable means of transport, including active modes of transport (4%), carpooling, and using public transport. The remaining students used cars or motorcycles. In this same study, the authors found differences between the various university campuses [23]. In the districts that were farthest from the city, students preferred to use private means of transport in comparison with the campuses located near the city centre or the historical centre, whose students preferred to use public means of transport [23]. Similar differences between campuses have also been found in our study. In a study with UCLA students (USA), 32.7% of the students went to campus by car or motorcycle only, 30.9% used public transportation, 8.5% travelled by carpool, and 24.8% of the students went to their study centre on foot or by bicycle [28].

In reference to the motivation shown by the students for the choice of various means of transport, a study by the University of Bergamo showed that students gave greater importance to comfort followed by sustainability and safety [23]. Ramakreshnan et al. (2020) studied the motivation of the university community at a tropical university in Kuala Lumpur, Malaysia, for walking on campus [24]. The reasons stated included the proximity of the buildings (90.7%), as exercise (88.7%), for transport (72.8%), and as recreation (55.6%) [24].

Universities have a certain ethical commitment to society and can contribute through their way of promoting the construction of a more sustainable society [13,14,15,19]. Universities train the professionals and citizens of the future: in their classrooms, policymakers are formed [27,51,52]. In this respect, it is crucial that universities address sustainability.

There are multiple possibilities for the development of the SDGs in the university context [13]: the implementation of transformative actions can provide a good opportunity to contribute towards the construction of a more sustainable society [27]. One of the main problems in the implementation of the Sustainable Development Goals is their multidisciplinary nature. In order to achieve these goals, it is necessary for the various actors to work together [11]. Universities can contribute towards sustainability in a double direction, on the one hand by reducing the negative impacts they produce, and on the other hand by promoting improvement actions that make it possible to build a more sustainable world [27].

Mobility carries a very important weight in the study of university life cycle analysis [19]. In a study of the ecological footprint of the University Campus of Politecnico di Torino (Italy), it was determined that 52.7% of the carbon footprint was due to mobility [53]. Other studies have corroborated the importance of mobility in the analysis of the life cycle of universities as in the Polytechnic University of Valencia [54] and the University of Leon (Vegazana Campus) [55].

It has been studied how the users themselves see their means of transport in the future.

On the one hand, the vehicles that the respondents themselves consider will be used more in the future (with respect to the means of transport they currently use) are the scooter (both electric and conventional), the metro, and the bicycle. On the other hand, those that will be used the least are the ICE car, the bus, and walking.

The electric scooter has undoubtedly been one of the revolutions of the 2019–2020 academic year (first lockdown in Spain), which has changed the current panorama in cities to the point of legislation having to be created for its control [56]. This type of mobility is still growing in popularity in Seville since it is a flat city, the weather is good throughout the year, and there is already a good cycling network, which is where these users are usually observed. For these reasons, the bicycle will continue to perform better in the future according to the respondents. The university in Seville considers that the metro will be the best transport in the future after the scooter, any many wish that the metro lines would reach the HTSE so that cars and buses could be done without.

As for those that will be used the least, the university in Seville is aware that the ICE car is something of the past, and they do not expect this type of vehicle to continue in use in the future. The same happens with travelling on foot, since it is considered not very comfortable and very slow, and there are already options on the market, such as the bicycle and the electric scooter, that are not only more comfortable and faster but are also low-polluting vehicles. Finally, the bus is a somewhat outdated vehicle according to the opinion of the students, because it is a community, public, and slow means of transport, and it is not popular among student users.

Today, society is embroiled in a global problem, the coronavirus pandemic, which has direct implications regarding daily transportation. Nowadays, people generally create overprotective measures to prevent themselves from becoming infected with the virus, and hence solo transport and private vehicles have increased in number while normal and public means of transport have decreased [57].

Furthermore, in public vehicles, such as the bus or the metro, the mandatory use of masks and a safe distance should be maintained at all times, which leads to fewer people allowed on buses. However, if before the pandemic the buses were full at peak times to the point that it would be necessary to increase the fleet, it will now be necessary to calculate whether this fleet should be increased, since fewer people fit on the bus, while at the same time, there are fewer people travelling by public transport. This in turn will affect the emission factor of buses during the pandemic, since there will be fewer people inside them, and therefore more pollutant load will be distributed per person.

Another type of mobility that is more in demand every year is that of vehicle-sharing systems: public bicycles, rental electric scooters, and rental electric motorbikes. However, it is a difficult period for these types of companies due to the fact that people have become more distrustful of car-sharing systems.

The current paradigm is undergoing a changing process; no one knows with certainty what will happen with the type of mobility that we are going to have, but it is believed that the trend towards sustainability will continue to grow.

In order to study the type of student mobility, one major requirement involves ascertaining whether university students consider sustainability to be relevant regarding their transportation.

When answering whether they could change to a more sustainable means of transport, the majority (44.34%) without any doubt answered yes, another large part (27.30%) had doubts and answered that perhaps they would, and another large part (28.35%) stated that they would not change.

In this work, we have studied the feelings that these means of transport transmit to the university students surveyed. The electric car conveys sustainability and comfort; the bicycle, on the other hand, conveys freedom and sportsmanship; the metro triggers feelings of speed, sustainability, comfort, and boredom; the electric motorcycle signifies freedom and speed; and the scooter conveys freedom and comfort.

In order to analyse the user, an initial study of age, gender, and the most frequent degrees and masters, among others, has been carried out. Various classes of user are distinguished by carrying out a user test, thereby obtaining clusters or groups of users. Seven user profiles have been obtained from a cluster analysis, and profiles have been generated according to the data obtained.

Regarding the results of the ecological footprint caused by the university student, two relevant results are extracted.

First, the mode or means of transportation used by the engineering student is discussed. More than one in four students decide to travel by private car to make the journey, while more than one in four students choose to catch the bus. These two are the most commonly used means of transport, respectively. On the other hand, combining walking with cycling also accounts for just over a quarter of the total, and it even exceeds the proportion travelling by bus.

Second, the results obtained after their analysis are examined. As expected, more kg of CO_2_ are emitted per week per person in the HTSE than in the HPS: 10.54 compared to 8.36. In addition, the HTSE has more students than the HPS, and hence, in terms of final emissions, it is normal for them to be higher than 1896.91 tons compared to 702.44.

Currently, the two centres together emit 2600 tons of CO_2_, which is a major figure to take into account, since only students and their transport are being counted. In comparison with other Spanish universities, the tons emitted are similar; it can be compared, for example, with the Campus Vegazana of the University of León, which, in 2006, polluted 1425.26 tons of CO_2_ through cars [55], with around 10,000 students. Approximately, the HTSE and the HPS account for 30% of the total by car, which would be 780 tons of CO_2_. In addition, knowing that the total number of students is 8800, it can be seen that it is a somewhat lower figure than that of the Vegazana Campus, so it can be said that the result is optimal.

On the other hand, if the university is observed in its entirety, in 2007, the University of Santiago de Compostela emitted 5749.80 tons of CO_2_ [10] with 30,200 students, and in 2011, the University of Valencia emitted 32,562.10 tons of CO_2_ with more than 60,000 people [58].

As can be observed, the transfer of the Higher Polytechnic School will incur 25.9% more impact on CO_2_ when comparing the two locations, since it will increase from 702.44 tons of CO_2_/year to 885.22. This is what will be assumed in the transfer of the location of the polytechnic from its present location in Los Remedios to the new premises in Cartuja. However, as there are fewer students than in the Higher Technical School of Engineers, it remains less polluted than the latter.

In our case, most of the CO_2_ emissions are due to the use of the ICE car, which is why we should encourage the use of less polluting vehicles and mainly means of transport with zero real emissions (bicycle and walking). Although the electric vehicle does not directly emit CO_2_, the CO_2_ needed to produce the electricity it consumes should be taken into consideration.

There are some experiences of promoting sustainable transport in universities in the context of the process of transformation towards the so-called green campuses [27,59]. In this respect, the University of Florence provided students with a card for public transport at a reduced price [27]. This same university joined national and international networks for the promotion of sustainable mobility [27] such as the European Network for Sustainable Mobility at Universities [60]. This network is the result of a project co-financed by the European Union that is being developed in the 2016–2021 timeframe and seeks to promote sustainable modes of transport in the context of European universities [60].

In the case of Qatar university, Azzali and Abdel Sabour (2018) provide several proposals for the improvement of mobility: improve bus lines for students, create bus lines for staff living in the same compound, promote transportation of a more sustainable nature among the university community, increase teleworking, and create public transportation infrastructure, such as metros and railroads [1]. Chulalongkorn University proposed several actions that include the creation of a parking system that reduces the use of the car within the campus, by promoting collective means of transport, the electric vehicle, and also the bicycle and travelling on foot [2]. Previous work has found barriers to the use of sustainable means of transport such as bicycles, including perceived safety, the need for helmets, low awareness, and physical fitness [18].

The reasons most often given in student responses for making a change in modes of transportation are speed, sustainability, and economics. Therefore, means of transport that include these three characteristics should be encouraged. Given the circumstances brought about by the current COVID-19 pandemic, light vehicles, such as bicycles and scooters, should probably be considered.

## 5. Conclusions

Universities play a crucial role in promoting sustainability and achieving SDGs. Higher education institutions can contribute towards these objectives through all their functions (teaching, research, transfer) and even through their operation, thereby contributing to the construction of a better society.

The interurban mobility of engineering students from two centres of the University of Seville has been studied. Students from each of the study centres use various means of transportation. Three constructs could be found to describe the motivation for choosing the means of transport. The first of these includes factors such as speed, ease of access, freedom, and comfort. The second is related to sustainability and the price of the means of transport. The last of these factors is safety.

In general, students are satisfied with the most frequently used means of transportation. Users give lower marks to collective means of transport (train, bus, and metro).

Students who use transportation of a more polluting character show a desire to switch to another means of transportation. A greater fidelity to the chosen means of transport was found among the students who use low-emission means of transport. The most frequently chosen alternative means of transportation was the electric car followed by the bicycle. Based on the students’ answers, seven student profiles have been found through the application of clustering. These groups of students would allow the design of specific actions according to each of the profiles.

Regarding CO_2_ emissions, the average values of the two university centres differ. Most of the emissions are due to the use of ICE cars. This shows a direction in which to work towards reducing emissions in urban mobility among these students.

The conclusions of this study cover both the emotions that the modes and means of transport produce in engineering students and the calculation of CO_2_ emissions that they produce due to their displacement.

Specifically, the conclusions are established in four areas:

1. Each mode or means of transport conveys a different emotion.
2. There is a difference in the type of mobility depending on the location of the university centre.
3. The transfer of the HPS to Cartuja will incur an increase in CO_2_ emissions.
4. Despite the differences between the centres and their mobility style, both HPS and HTSE students measure each emotion in a similar way, except regarding sustainability and economy.

The university student considers it crucial that the ideal mode or means of transport should be a fast and comfortable vehicle, which grants a feeling of freedom, that is, one that feels free at the time of use and that removes any type of burden such as that caused by heavy traffic or the lack of convenient parking spaces. Furthermore, it must also be a sustainable and economical mode or means of transport.

In general terms, no priority is placed on the safety or quietness of the vehicle, nor is autonomy a requirement, as long as the means is sufficient to travel from home to school and vice versa.

The best-valued modes and means of transport are light, among which are both the bicycle and the scooter.

The three ideal vehicles for engineering university students are the bicycle, the scooter, and the motorcycle, since they are economical vehicles, fast in the city, comfortable both when moving around and avoiding traffic jams and when parking quickly; they offer a feeling of freedom, and they are relatively sustainable (the bicycle being more sustainable than the motorcycle). However, for students who feel that they need a safer vehicle, they are likely to choose other means, such as the car or walking. In addition, there are students who live far from the school and travel by public transport or by car, but they take it as the only option, not as the one that best meets their needs.

Possible future work is proposed both in the field of sustainability and for Industrial Design and Product Development.

In the line of sustainability, it is proposed to continue this line of reasoning and find the ecological footprint of the University of Seville. The electric scooter and the subway are the means of transport that the students consider that they will be more used in the future. This is the reason why a comparative study has been carried out between these two means of transport using Kansei Engineering. On the other hand, more than one-third of those surveyed use the car individually, which is the most polluting means of transport. The possibility of modeling an interuniversity Carsharing APP or web page is proposed to help reduce emissions. Finally, a line that is closer to marketing could involve the creation of a communication strategy to make university youth aware of the importance of using transport of a more sustainable nature.

Based on the results obtained, the recommendations for policymakers and a reflection on the impact of COVID-19 on university mobility are set out below.

### 5.1. Recommendations for Policymakers

Considering the results of the work, the following recommendations could be proposed to policymakers.

1. Institutions of higher education should be evaluated using instruments that go beyond their research activity [10]. The activity of universities is oriented towards the position and impact on the rankings [61]. In this respect, it is fundamental that parameters of sustainability and compliance with the SDGs begin to appear in these rankings [7,61], and they should be considered for the financing of the universities [16]. The creation of rankings that evaluate the role of higher education institutions in achieving the SDGs, such as “The Impact Ranking” [62], is an opportunity for higher education institutions [7]. In the second edition of this ranking, 768 universities from 85 countries were evaluated, and the results were measured in three areas: research, outreach, and stewardship [62].
2. The role of sustainability offices or green offices needs to be strengthened to promote change in universities [14,63]. Recent work has pointed out the lack of funds, the lack of support from the administration [63], and the lack of interest from students and staff as possible barriers for these institutions. In this respect, the incorporation of green offices that are led by students will promote the construction of a more sustainable university. In this way, the role of students remains highly significant, and their opinions must be taken into account as a starting point for future policies and investments in the university [18].
3. In order to promote sustainable means of transport, it is essential to create an ecosystem that makes transport comfortable, fast, and safe. In this respect, initiatives such as bike sharing [2,59] and a cycle path system can contribute towards the promotion of these means of transport among university students. It is also interesting to promote these means of transport through the use of subsidies and the improvement of collective means of transport [27,28].

### 5.2. Universities in the Post-COVID Era

The surveys were applied before the first pandemic lockdown. One of the future lines of work will be to study the variation in student opinion due to the presence of a disease, such as COVID-19, which is transforming the way classes are taught [64,65,66] and altering mobility patterns in our cities [67].

In post-pandemic conditions, the perception of public transportation as unhealthy can gain ground and remain. It is necessary to restore the capacity of public transport systems to fulfill their social role [68].

Before the pandemic, transport policy was focused on demand management, “smart” technology interventions, and sustainable mobility. The situation resulting from the pandemic presents an opportunity to reconfigure future transportation policies and practices, with an environmental perspective, both from a global point of view and by individual citizens. This public health crisis has required a new vision of transportation and its role in economic recovery [69].

Many cities have chosen to promote collective transport to reduce pollution [2,59]. However, the existence of an illness that requires an increase in social distancing can make these means of transport more unsafe. In this regard, it is necessary to take extreme measures to make public transport a safe place, one that allows social distancing to be maintained, in order not to harm its users [57]. The results presented in this work should be contrasted with future research regarding the opinions of users who are aware of the risk of the disease.

University education in the aftermath of the COVID-19 pandemic will need to be even more focused on the development of student skills towards active learning [64]. In the current context, there is a trend towards the digitalisation of universities and teleworking [65,66]. Obviously, these circumstances will exert a positive effect on mobility, and therefore, in turn, its effect on the ecological footprint of the university should be studied. Azzali and Abdel Sabour proposed telecommuting as a tool to improve the mobility of the University of Qatar in 2018 [1].

Although the work was developed prior to the pandemic containment, it provides a baseline for measuring future changes in student mobility behaviour.

## Figures and Tables

**Table 1:** CO_2_ emissions per kilometre for the internal combustion engine (ICE) car [40] and the hybrid car [41] as a function of occupancy.

	Occupancy Level			
	25% (1 or 2 People)	50% (3 People)	75% (4 People)	100% (5 People)
ICE car [kg of CO_2_/km]	0.2	0.1	0.07	0.05
Hybrid car [kg of CO_2_/km]	0.11	0.07	0.05	-

**Table 2:** Average student responses to items Q7–Q14 (motivation for choice of transportation).

		Q7	Q8	Q9	Q10	Q11	Q12	Q13	Q14
Walking	HTSE	3.31	4.65	4.58	3.85	4.85	4.88	4.92	4.08
	HPS	3.51	4.55	4.55	3.89	4.83	4.87	4.87	4.10
	Total	3.45	4.58	4.56	3.88	4.84	4.88	4.89	4.09
Bus	HTSE	2.87	4.22	2.77	2.93	3.68	3.90	2.64	3.84
	HPS	2.75	4.31	2.58	3.07	3.67	3.58	2.29	3.71
	Total	2.82	4.26	2.70	2.99	3.68	3.78	2.51	3.79
Bicycle	HTSE	4.15	4.65	4.50	4.00	4.92	4.85	4.92	3.50
	HPS	3.97	4.65	4.59	3.89	5.00	4.89	4.89	3.41
	Total	4.05	4.65	4.56	3.94	4.97	4.87	4.90	3.44
Car	HTSE	4.29	4.66	4.46	4.73	2.24	2.91	1.42	3.73
	HPS	4.08	4.76	4.48	4.62	2.20	2.84	1.50	3.58
	Total	4.23	4.69	4.47	4.70	2.23	2.89	1.45	3.69
Metro	HTSE	3.29	3.57	2.43	2.71	3.29	2.29	2.29	4.00
	HPS	4.03	4.51	3.00	3.36	3.85	2.87	3.33	4.05
	Total	3.91	4.37	2.91	3.26	3.76	2.78	3.17	4.04
Motorcycle	HTSE	4.25	4.88	4.75	4.13	2.88	4.25	2.00	2.63
	HPS	4.48	4.87	4.48	4.04	2.78	3.96	1.65	2.57
	Total	4.42	4.87	4.55	4.06	2.81	4.03	1.74	2.58
Skateboard	HTSE	4.67	4.33	4.67	4.67	4.33	4.67	4.33	3.67
	HPS	4.43	4.86	4.86	4.57	4.29	4.71	4.29	3.14
	Total	4.50	4.70	4.80	4.60	4.30	4.70	4.30	3.30
Train	HTSE	3.33	3.67	2.33	2.83	3.50	2.83	3.00	3.67
	HPS	4.50	4.75	4.00	3.75	4.50	3.00	4.00	4.75
	Total	3.80	4.10	3.00	3.20	3.90	2.90	3.40	4.10

**Table 3:** Results of normality tests and differences between simples (ANOVA and non-parametric test).

			Q7	Q8	Q9	Q10	Q11	Q12	Q13	Q14
Walking	HTSE	Sig. ^1^	0.002	0.000	0.000	0.001	0.000	0.000	0.000	0.000
	HPS	Sig. ^2^	0.000	0.000	0.000	0.000	0.000	0.000	0.000	0.000
	Dif.	Sig. ^3^	0.378 ^U^	0.550 ^U^	0.652 ^U^	0.935 ^U^	0.628 ^U^	0.680 ^U^	0.578 ^U^	0.756 ^U^
Bus	HTSE	Sig. ^2^	0.000	0.000	0.000	0.000	0.000	0.000	0.000	0.000
	HPS	Sig. ^2^	0.000	0.000	0.000	0.000	0.000	0.000	0.000	0.000
	Dif.	Sig. ^3^	0.439 ^U^	0.529 ^U^	0.238 ^U^	0.352 ^U^	0.914 ^U^	0.086 ^U^	0.044 ** ^U^	0.423 ^U^
Bicycle	HTSE	Sig. ^1^	0.000	0.000	0.000	0.000	0.000	0.000	0.000	0.002
	HPS	Sig. ^1^	0.000	0.000	0.000	0.000	0.000	0.000	0.000	0.000
	Dif.	Sig. ^3^	0.395 ^U^	0.803 ^U^	0.555 ^U^	0.697 ^U^	0.089 ^U^	0.398 ^U^	0.929 ^U^	0.652 ^U^
Car	HTSE	Sig. ^2^	0.000	0.000	0.000	0.000	0.000	0.000	0.000	0.000
	HPS	Sig. ^2^	0.000	0.000	0.000	0.000	0.000	0.000	0.000	0.000
	Dif.	Sig. ^3^	0.129 ^U^	0.526 ^U^	0.928 ^U^	0.138 ^U^	0.682 ^U^	0.658 ^U^	0.679 ^U^	0.248 ^U^
Metro	HTSE	Sig. ^1^	0.482 *	0.609 *	0.020	0.183 *	0.086 *	0.086 *	0.086 *	0.144 *
	HPS	Sig. ^2^	0.000	0.000	0.001	0.000	0.000	0.000	0.000	0.000
	Dif.	Sig. ^3^	0.068 ^U^	0.006 ** ^U^	0.152 ^U^	0.088 ^U^	0.079 ^U^	0.162 ^U^	0.009 ** ^U^	0.792 ^U^
Motorcycle	HTSE	Sig. ^1^	0.000	0.000	0.000	0.001	0.006	0.018	0.030	0.324 *
	HPS	Sig. ^1^	0.000	0.000	0.000	0.000	0.000	0.001	0.000	0.014
	Dif.	Sig. ^3^	0.677 ^U^	0.969 ^U^	0.318 ^U^	0.681 ^U^	0.921 ^U^	0.332 ^U^	0.327 ^U^	0.831 ^U^
Skateboard	HTSE	Sig. ^1^	0.000	0.000	0.000	0.000	0.000	0.000	0.000	0.000
	HPS	Sig. ^1^	0.001	0.000	0.000	0.001	0.001	0.000	0.008	0.006
	Dif.	Sig. ^3^	0.513 ^U^	0.416 ^U^	0.513 ^U^	0.789 ^U^	1.000 ^U^	0.886 ^U^	0.896 ^U^	0.398 ^U^
Train	HTSE	Sig. ^1^	0.000	0.001	0.091 *	0.421 *	0.101	0.804 *	0.960	0.473
	HPS	Sig. ^1^	0.024	0.001	0.683 *	0.001	0.024	0.683 *	0.024	0.001
	Dif.	Sig. ^3^	0.037 ^U^	0.021 ^U^	0.013 ** ^A^	0.166 ^U^	0.165 ^U^	0.844 ^A^	0.265 ^U^	0.069 ^U^

^1^*p*-value for normality test (Shapiro–Wilk test, in bold normal samples) ^2^*p*-value for normality test (Kolmogorov–Smirnov test, * normal samples) ^3^*p*-value ANOVA test and Mann–Whitney U test, in ** significative differences, ^A^ ANOVA, ^U^ Mann–Whitney U test.

**Table 4:** Kaiser–Meyer–Olkin (KMO) and Bartlett test.

Kaiser–Meyer–Olkin Measurement of Sampling Adequacy		0.697
Bartlett Sphericity Test	χ²	1768.002
	Degrees of freedom	28
	Sig.	0.000

**Table 5:** Eigenvalues and variance explained.

Component	Eigenvalue	% Explained Variance	% Explained Accumulated Variance
1	2.548	31.856	31.856
2	2.340	29.256	61.111
3	1.002	12.520	73.631
4	0.693	8.664	82.295
5	0.550	6.872	89.167
6	0.401	5.017	94.184
7	0.300	3.747	97.931
8	0.166	2.069	100.000

**Table 6:** Rotated component matrix.

	Components		
	1	2	3
Q7	−0.111	0.751	−0.092
Q8	0.084	0.667	0.015
Q9	0.158	0.830	−0.057
Q10	−0.201	0.816	0.188
Q11	0.906	−0.115	0.165
Q12	0.843	0.114	−0.092
Q13	0.903	−0.033	0.122
Q14	0.121	0.015	0.977

**Table 7:** Results for Kolmogorov–Smirnov test.

	Group	Statistic	Degree of Freedom	Sig.
Q15	CPT	0.257	376	0.000
	NC	0.230	200	0.000
Q16	CPT	0.182	376	0.000
	NC	0.215	200	0.000

**Table 8:** Results for non-parametric tests.

	Q15	Q16
Mann–Whitney U	16,206.500	30,917.000
Wilcoxon W	36,306.500	101,793.000
Z	−12.056	−3.618
Asym. Sig. (2-tailed)	0.000	0.000

**Table 9:** Percentage of students who would change their mode of transport to another sustainable mode.

Current Mode of Transport	Yes	Maybe	No
Walking	23%	17%	60%
Bus	55%	34%	11%
Bicycle	8%	19%	73%
Car	65%	23%	12%
Metro	32%	50%	12%
Motorbike	36%	48%	16%
Skateboard/scooter	62%		38%
Train	62%	13%	25%

**Table 10:** Absolute frequency of reasons for changing means of transport (Q19.2).

Reason	Frequency
Comfort	40
Rapidity	75
Economy	42
Sustainability	45
Calm	10

**Table 11:** Final cluster centres: seven user profiles.

	Cluster						
	1	2	3	4	5	6	7
Transport	2	4	3	5	8	1	5
Satisfaction	2.89	4.14	4.53	3.68	4.62	4.02	2.09
Future	2.18	2.05	4.08	4.16	3.49	3.47	2.36
Sustainable Change	2	1	2	2	2	2	1
Average	3.24	3.52	4.22	3.67	3.83	4.00	2.91

**Table 12:** Number of students associated with each cluster.

Cluster	Number of Students
1	83
2	145
3	90
4	50
5	39
6	158
7	11

## Appendix A

**Table A1:** Questionnaire.

Question Number	Question Text	Variable Type	Options
Q1	Gender	Categorical	Male
			Female
			Prefer not to say
Q2	Age	Quantitative	
Q3	Type of academic programme	Categorical	Bachelor’s degree
			Master’s degree
Q4	Name of the degree	Open	
Q5	Habitual home address	Open	
Q6	Most commonly used means of transport	Categorical	Walking
			Bicycle
			Scooter/skateboard
			Metro
			Bus
			Motorcycle
			Car
			Other
Q6.1	Type of bicycle (If Q6 = Bicycle)	Categorical	Public
			Electric bicycle
			Conventional
Q6.2	Type of scooter/skateboard (If Q6 = scooter/skateboard)	Categorical	Private electric scooter
			Private conventional scooter
			Rental scooter
			Electric skateboard
			Conventional skateboard
Q6.3	Number of bus (if Q6 = bus)	Open	
Q6.4.1	Type of motorcycle (if Q6 = motorcycle)	Categorical	Private electric motorcycle
			Private ICE motorcycle
			Rental electric motorcycle
			Rental ICE motorcycle
Q6.4.2	Do you share the motorcycle? (if Q6 = motorcycle)	Categorical	Yes
			Usually
			Rarely
			No
Q6.5.1	Type of car (if Q6 = car)	Categorical	Private electric car
			Private ICE car
			Private hybrid car
			Rental electric car
			Rental ICE car
			Rental hybrid car
Q6.5.2	Do you share the car? (if Q6 = car)	Categorical	Yes
			Maybe
			No
Q6.5.3	Number of passengers	Quantitative	
**Indicate Your Degree of Agreement/Disagreement About the Most Frequently Used Means of Transport (Q7–Q16)**			
Q7	This means of transport is fast.	Ordinal	Likert scale (1–5) ^1^
Q8	I can easily access my most usual means of transport.	Ordinal	Likert scale (1–5) ^1^
Q9	This means of transport makes me feel free.	Ordinal	Likert scale (1–5) ^1^
Q10	This means of transport is comfortable.	Ordinal	Likert scale (1–5) ^1^
Q11	This means of transport is sustainable.	Ordinal	Likert scale (1–5) ^1^
Q12	This means of transport is cheap.	Ordinal	Likert scale (1–5) ^1^
Q13	This means of transport does not pollute.	Ordinal	Likert scale (1–5) ^1^
Q14	This means of transport is safe.	Ordinal	Likert scale (1–5) ^1^
Q15	My degree of satisfaction with this means of transport is…	Ordinal	Likert scale (1–5) ^2^
Q16	In the future, this means of transport will be used more than at present.	Ordinal	Likert scale (1–5) ^1^
Q17	Would you change your means of transport to a more sustainable one?	Categorical	Yes
			Maybe
			No
Q18	To which one?	Categorical	Walking
			Bicycle
			Scooter/skateboard
			Metro
			Bus
			Electric motorcycle
			Electric car
			Other:
Q19.1	What does the chosen means of transport do to you emotionally? (if Q17 = Yes)	Open	
Q19.2	What should the new means of transport be like in order to bring about change? What feeling would it convey? (If Q17 = Maybe)	Open	

^1^ (1) Strongly disagree, (5) Strongly agree. ^2^ (1) Very low, (5) Very high.
